# 1,2-Diphenyl-2-[4-(4-pyridyl)benzyl­idene­hydrazono]ethan-1-one

**DOI:** 10.1107/S1600536809026087

**Published:** 2009-07-11

**Authors:** Goutam Kumar Patra, Seik Weng Ng

**Affiliations:** a Department of Chemistry, Vijaygarh Jyotish Ray College, 8/2 Vijaygarh, Jadavpur, Kolkata 700 032, India; b Department of Chemistry, University of Malaya, 50603 Kuala Lumpur, Malaysia

## Abstract

In the title compound, C_26_H_19_N_3_O, the dimethyl­ene hydrazine (—C=N—N=C—) unit is approximately planar, the torsion angle around the N—N bond being 162.2 (6)°. The phenyl and benzoyl­phenyl rings at one end of the hydrazine unit are aligned at angles of 9.5 (5) and 88.5 (4)°, respectively, with respect to the hydrazine unit, whereas the benzene ring at the other end is twisted by an angle of 14.4 (4)°. In the crystal structure, mol­ecules are linked into centrosymmetric dimers by inter­molecular C—H⋯O hydrogen bonds. The monoclinic crystal under investigation shows pseudo-merohedral twinning with twin fractions of 0.63 and 0.37.

## Related literature

For the crystal structures of other carbaldehyde *N*′-benzoyl-*N*′-phenyl­hydrazones, see: Abbasi *et al.* (2007 ▶); Chowdhury *et al.* (2003 ▶); Liu *et al.* (2007 ▶); Schweizer *et al.* (1987 ▶).
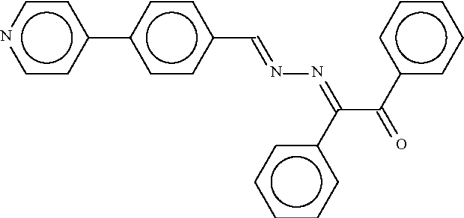


## Experimental

### 

#### Crystal data

C_26_H_19_N_3_O
*M* *_r_* = 389.44
Monoclinic, 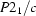

*a* = 7.1182 (2) Å
*b* = 23.2745 (7) Å
*c* = 11.8040 (4) Å
β = 90.278 (2)°
*V* = 1955.6 (1) Å^3^
*Z* = 4
Mo *K*α radiation
μ = 0.08 mm^−1^
*T* = 140 K
0.45 × 0.15 × 0.05 mm

#### Data collection

Bruker SMART APEX area-detector diffractometer
Absorption correction: none
11057 measured reflections
3433 independent reflections
2825 reflections with *I* > 2σ(*I*)
*R*_int_ = 0.054

#### Refinement

*R*[*F* ^2^ > 2σ(*F* ^2^)] = 0.093
*wR*(*F* ^2^) = 0.252
*S* = 1.08
3433 reflections
260 parameters
H-atom parameters constrained
Δρ_max_ = 0.63 e Å^−3^
Δρ_min_ = −0.38 e Å^−3^

### 

Data collection: *APEX2* (Bruker, 2008 ▶); cell refinement: *SAINT* (Bruker, 2008 ▶); data reduction: *SAINT*; program(s) used to solve structure: *SHELXS97* (Sheldrick, 2008 ▶); program(s) used to refine structure: *SHELXL97* (Sheldrick, 2008 ▶); molecular graphics: *X-SEED* (Barbour, 2001 ▶); software used to prepare material for publication: *publCIF* (Westrip, 2009 ▶).

## Supplementary Material

Crystal structure: contains datablocks global, I. DOI: 10.1107/S1600536809026087/ci2837sup1.cif

Structure factors: contains datablocks I. DOI: 10.1107/S1600536809026087/ci2837Isup2.hkl

Additional supplementary materials:  crystallographic information; 3D view; checkCIF report

## Figures and Tables

**Table 1:** Hydrogen-bond geometry (Å, °)

*D*—H⋯*A*	*D*—H	H⋯*A*	*D*⋯*A*	*D*—H⋯*A*
C26—H26⋯O1^i^	0.95	2.57	3.502 (7)	166

Symmetry code: (i) 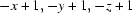.

**Fig. 1.**
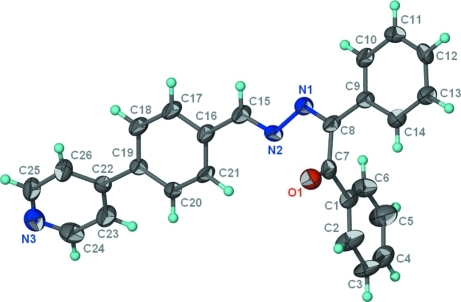
Displacement ellipsoid plot (Barbour, 2001) of C26H19N3O at the 70% probability level; H atoms are drawn as spheres of arbitrary radius.

## supplementary crystallographic information

### Experimental

Benzil monohydrazone (0.224 g, 1 mmol) was dissolved in methanol (20 ml) and to this was added 4-pyridylbenzaldehyde (0.183 g, 1 mmol). The resulting yellowish mixture was heated for 6 h. The solvent was evaporated and the solid was recrystallized from methanol in 80% yield; m.p. 461 K.

### Refinement

H atoms were placed in calculated positions (C-H = 0.95 Å) and were included in the refinement in the riding model approximation, with *U*(H) set to 1.2*U*(C). The aromatic ring of the benzoyl unit was refined as a rigid hexagon (C—C = 1.39 Å); attempts to refine the ring as two overlapping rings were unsuccessful. The monoclinic unit cell emulates an orthorhombic unit cell; the use of the twin law (-100 010 001) showed twin fractions are in the ratio 0.63:0.37.

### Crystal data

**Table:**

C_26_H_19_N_3_O	*F*(000) = 816
*M**_r_* = 389.44	*D*_x_ = 1.323 Mg m^−^^3^
Monoclinic, *P*2_1_/*c*	Mo *K*α radiation, λ = 0.71073 Å
Hall symbol: -P 2ybc	Cell parameters from 2404 reflections
*a* = 7.1182 (2) Å	θ = 2.5–23.3°
*b* = 23.2745 (7) Å	µ = 0.08 mm^−^^1^
*c* = 11.8040 (4) Å	*T* = 140 K
β = 90.278 (2)°	Prism, brown
*V* = 1955.6 (1) Å^3^	0.45 × 0.15 × 0.05 mm
*Z* = 4	

### Data collection

**Table:**

Bruker SMART APEX area-detector diffractometer	2825 reflections with *I* > 2σ(*I*)
Radiation source: fine-focus sealed tube	*R*_int_ = 0.054
graphite	θ_max_ = 25.0°, θ_min_ = 0.9°
ω scans	*h* = −8→8
11057 measured reflections	*k* = −27→27
3433 independent reflections	*l* = −13→14

### Refinement

**Table:**

Refinement on *F*^2^	Primary atom site location: structure-invariant direct methods
Least-squares matrix: full	Secondary atom site location: difference Fourier map
*R*[*F*^2^ > 2σ(*F*^2^)] = 0.093	Hydrogen site location: inferred from neighbouring sites
*wR*(*F*^2^) = 0.252	H-atom parameters constrained
*S* = 1.08	*w* = 1/[σ^2^(*F*_o_^2^) + (0.1142*P*)^2^ + 4.6222*P*] where *P* = (*F*_o_^2^ + 2*F*_c_^2^)/3
3433 reflections	(Δ/σ)_max_ = 0.001
260 parameters	Δρ_max_ = 0.63 e Å^−^^3^
0 restraints	Δρ_min_ = −0.38 e Å^−^^3^

### Special details

**Table:**

Geometry. All e.s.d.'s (except the e.s.d. in the dihedral angle between two l.s. planes) are estimated using the full covariance matrix. The cell e.s.d.'s are taken into account individually in the estimation of e.s.d.'s in distances, angles and torsion angles; correlations between e.s.d.'s in cell parameters are only used when they are defined by crystal symmetry. An approximate (isotropic) treatment of cell e.s.d.'s is used for estimating e.s.d.'s involving l.s. planes.

### Fractional atomic coordinates and isotropic or equivalent isotropic displacement parameters (Å^2^)

**Table:**

	*x*	*y*	*z*	*U*_iso_*/*U*_eq_	
O1	0.5484 (6)	0.38713 (17)	0.8664 (3)	0.0404 (10)	
N1	0.2040 (9)	0.37367 (17)	0.7012 (4)	0.0419 (13)	
N2	0.2387 (8)	0.43390 (17)	0.6961 (3)	0.0343 (11)	
N3	0.2956 (9)	0.8061 (2)	0.3966 (4)	0.0487 (15)	
C1	0.2861 (6)	0.41267 (14)	0.9747 (2)	0.0366 (15)	
C2	0.3933 (5)	0.4384 (2)	1.0593 (3)	0.0541 (18)	
H2	0.5266	0.4374	1.0554	0.065*	
C3	0.3056 (6)	0.46560 (18)	1.1495 (3)	0.0542 (18)	
H3	0.3789	0.4832	1.2074	0.065*	
C4	0.1107 (6)	0.46707 (17)	1.1552 (3)	0.0477 (17)	
H4	0.0507	0.4857	1.2169	0.057*	
C5	0.0034 (5)	0.4413 (2)	1.0705 (4)	0.0566 (19)	
H5	−0.1298	0.4424	1.0744	0.068*	
C6	0.0911 (5)	0.41414 (17)	0.9803 (3)	0.0427 (15)	
H6	0.0178	0.3966	0.9224	0.051*	
C7	0.3789 (9)	0.3850 (2)	0.8787 (4)	0.0323 (13)	
C8	0.2589 (8)	0.3512 (2)	0.7921 (4)	0.0276 (11)	
C9	0.2362 (8)	0.2882 (2)	0.8099 (4)	0.0263 (11)	
C10	0.1549 (8)	0.2553 (2)	0.7251 (5)	0.0346 (13)	
H10	0.1065	0.2733	0.6589	0.041*	
C11	0.1442 (9)	0.1956 (2)	0.7373 (5)	0.0386 (14)	
H11	0.0916	0.1726	0.6787	0.046*	
C12	0.2116 (10)	0.1704 (2)	0.8361 (5)	0.0387 (14)	
H12	0.2102	0.1298	0.8435	0.046*	
C13	0.2799 (9)	0.2034 (2)	0.9232 (5)	0.0379 (14)	
H13	0.3164	0.1859	0.9925	0.045*	
C14	0.2956 (9)	0.2619 (2)	0.9104 (4)	0.0366 (14)	
H14	0.3470	0.2845	0.9700	0.044*	
C15	0.2301 (10)	0.4523 (2)	0.5948 (4)	0.0415 (16)	
H15	0.2082	0.4252	0.5360	0.050*	
C16	0.2517 (9)	0.5124 (2)	0.5636 (4)	0.0350 (13)	
C17	0.2450 (11)	0.5271 (2)	0.4494 (4)	0.0472 (18)	
H17	0.2366	0.4978	0.3937	0.057*	
C18	0.2504 (10)	0.5839 (2)	0.4160 (4)	0.0380 (14)	
H18	0.2482	0.5931	0.3377	0.046*	
C19	0.2593 (8)	0.62806 (19)	0.4964 (4)	0.0250 (11)	
C20	0.2708 (9)	0.6123 (2)	0.6106 (4)	0.0309 (12)	
H20	0.2821	0.6416	0.6665	0.037*	
C21	0.2661 (9)	0.5562 (2)	0.6441 (4)	0.0328 (13)	
H21	0.2727	0.5469	0.7224	0.039*	
C22	0.2679 (8)	0.6892 (2)	0.4604 (4)	0.0268 (11)	
C23	0.2038 (10)	0.7331 (2)	0.5287 (5)	0.0394 (15)	
H23	0.1455	0.7245	0.5990	0.047*	
C24	0.2249 (10)	0.7896 (2)	0.4943 (6)	0.0478 (16)	
H24	0.1852	0.8188	0.5451	0.057*	
C25	0.3562 (10)	0.7637 (2)	0.3315 (5)	0.0415 (15)	
H25	0.4118	0.7738	0.2613	0.050*	
C26	0.3452 (9)	0.7060 (2)	0.3573 (5)	0.0365 (13)	
H26	0.3898	0.6780	0.3054	0.044*	

### Atomic displacement parameters (Å^2^)

**Table:**

	*U*^11^	*U*^22^	*U*^33^	*U*^12^	*U*^13^	*U*^23^
O1	0.043 (3)	0.040 (2)	0.038 (2)	−0.0029 (19)	0.004 (2)	−0.0039 (18)
N1	0.079 (4)	0.018 (2)	0.029 (2)	−0.004 (2)	0.003 (3)	−0.0024 (18)
N2	0.058 (3)	0.0164 (19)	0.028 (2)	−0.002 (2)	−0.005 (2)	−0.0011 (16)
N3	0.071 (4)	0.030 (2)	0.045 (3)	−0.003 (3)	−0.005 (3)	0.007 (2)
C1	0.073 (5)	0.012 (2)	0.024 (2)	0.009 (3)	−0.003 (3)	0.0020 (18)
C2	0.054 (4)	0.074 (5)	0.035 (4)	0.005 (4)	0.000 (3)	−0.018 (3)
C3	0.084 (6)	0.053 (4)	0.026 (3)	−0.004 (4)	−0.001 (3)	−0.019 (3)
C4	0.073 (5)	0.038 (3)	0.033 (3)	0.008 (3)	0.006 (3)	0.000 (3)
C5	0.058 (5)	0.069 (5)	0.042 (4)	0.000 (4)	−0.002 (3)	−0.013 (3)
C6	0.047 (4)	0.040 (3)	0.041 (3)	0.002 (3)	0.000 (3)	−0.012 (3)
C7	0.052 (4)	0.019 (2)	0.026 (3)	0.000 (2)	0.001 (3)	0.008 (2)
C8	0.040 (3)	0.024 (2)	0.019 (2)	0.006 (2)	−0.001 (2)	−0.0001 (19)
C9	0.032 (3)	0.022 (2)	0.025 (2)	0.003 (2)	−0.002 (2)	−0.0011 (18)
C10	0.047 (4)	0.032 (3)	0.026 (3)	0.001 (3)	−0.001 (3)	0.006 (2)
C11	0.054 (4)	0.029 (3)	0.032 (3)	−0.006 (3)	0.000 (3)	−0.009 (2)
C12	0.060 (4)	0.018 (2)	0.038 (3)	−0.009 (3)	0.007 (3)	0.003 (2)
C13	0.054 (4)	0.032 (3)	0.028 (3)	−0.004 (3)	−0.002 (3)	0.000 (2)
C14	0.048 (4)	0.037 (3)	0.026 (3)	−0.006 (3)	0.002 (3)	−0.002 (2)
C15	0.084 (5)	0.018 (2)	0.022 (3)	0.007 (3)	0.005 (3)	−0.0030 (19)
C16	0.060 (4)	0.023 (2)	0.022 (2)	0.007 (3)	0.004 (3)	0.0016 (19)
C17	0.100 (6)	0.022 (3)	0.020 (2)	0.003 (3)	0.006 (3)	−0.005 (2)
C18	0.070 (4)	0.023 (2)	0.021 (2)	0.009 (3)	−0.001 (3)	−0.0002 (19)
C19	0.032 (3)	0.018 (2)	0.026 (2)	0.005 (2)	0.002 (2)	−0.0038 (18)
C20	0.047 (4)	0.022 (2)	0.025 (2)	−0.004 (2)	0.004 (3)	−0.0036 (19)
C21	0.052 (4)	0.031 (3)	0.015 (2)	0.002 (3)	−0.005 (3)	0.0019 (19)
C22	0.030 (3)	0.025 (2)	0.025 (2)	0.001 (2)	−0.004 (2)	0.0019 (18)
C23	0.058 (4)	0.030 (3)	0.029 (3)	0.008 (3)	0.009 (3)	0.000 (2)
C24	0.061 (4)	0.031 (3)	0.051 (4)	0.001 (3)	0.002 (4)	−0.011 (3)
C25	0.055 (4)	0.033 (3)	0.037 (3)	0.000 (3)	0.011 (3)	0.007 (2)
C26	0.043 (3)	0.035 (3)	0.031 (3)	0.003 (3)	0.007 (3)	0.005 (2)

### Geometric parameters (Å, °)

**Table:**

O1—C7	1.217 (7)	C12—C13	1.370 (8)
N1—C8	1.255 (7)	C12—H12	0.95
N1—N2	1.425 (6)	C13—C14	1.374 (8)
N2—C15	1.271 (7)	C13—H13	0.95
N3—C24	1.318 (8)	C14—H14	0.95
N3—C25	1.325 (8)	C15—C16	1.455 (7)
C1—C2	1.39	C15—H15	0.95
C1—C6	1.39	C16—C21	1.397 (7)
C1—C7	1.463 (6)	C16—C17	1.392 (7)
C2—C3	1.39	C17—C18	1.378 (7)
C2—H2	0.95	C17—H17	0.95
C3—C4	1.39	C18—C19	1.400 (7)
C3—H3	0.95	C18—H18	0.95
C4—C5	1.39	C19—C20	1.399 (7)
C4—H4	0.95	C19—C22	1.487 (6)
C5—C6	1.39	C20—C21	1.366 (7)
C5—H5	0.95	C20—H20	0.95
C6—H6	0.95	C21—H21	0.95
C7—C8	1.544 (8)	C22—C23	1.379 (7)
C8—C9	1.490 (7)	C22—C26	1.394 (7)
C9—C10	1.384 (7)	C23—C24	1.387 (8)
C9—C14	1.399 (7)	C23—H23	0.95
C10—C11	1.399 (8)	C24—H24	0.95
C10—H10	0.95	C25—C26	1.378 (8)
C11—C12	1.389 (8)	C25—H25	0.95
C11—H11	0.95	C26—H26	0.95
			
C8—N1—N2	113.1 (4)	C14—C13—H13	119.9
C15—N2—N1	111.3 (4)	C13—C14—C9	120.1 (5)
C24—N3—C25	114.6 (5)	C13—C14—H14	119.9
C2—C1—C6	120.0	C9—C14—H14	119.9
C2—C1—C7	119.8 (4)	N2—C15—C16	123.8 (5)
C6—C1—C7	120.1 (4)	N2—C15—H15	118.1
C1—C2—C3	120.0	C16—C15—H15	118.1
C1—C2—H2	120.0	C21—C16—C17	118.7 (4)
C3—C2—H2	120.0	C21—C16—C15	122.5 (4)
C4—C3—C2	120.0	C17—C16—C15	118.7 (5)
C4—C3—H3	120.0	C18—C17—C16	120.8 (5)
C2—C3—H3	120.0	C18—C17—H17	119.6
C5—C4—C3	120.0	C16—C17—H17	119.6
C5—C4—H4	120.0	C17—C18—C19	120.8 (5)
C3—C4—H4	120.0	C17—C18—H18	119.6
C4—C5—C6	120.0	C19—C18—H18	119.6
C4—C5—H5	120.0	C20—C19—C18	117.6 (4)
C6—C5—H5	120.0	C20—C19—C22	121.5 (4)
C5—C6—C1	120.0	C18—C19—C22	120.8 (4)
C5—C6—H6	120.0	C21—C20—C19	121.8 (4)
C1—C6—H6	120.0	C21—C20—H20	119.1
O1—C7—C1	121.7 (5)	C19—C20—H20	119.1
O1—C7—C8	119.2 (5)	C20—C21—C16	120.2 (4)
C1—C7—C8	119.1 (5)	C20—C21—H21	119.9
N1—C8—C9	119.8 (4)	C16—C21—H21	119.9
N1—C8—C7	121.5 (4)	C23—C22—C26	115.9 (5)
C9—C8—C7	117.9 (4)	C23—C22—C19	121.8 (4)
C10—C9—C14	119.7 (5)	C26—C22—C19	122.3 (5)
C10—C9—C8	119.2 (4)	C22—C23—C24	119.6 (5)
C14—C9—C8	121.1 (5)	C22—C23—H23	120.2
C11—C10—C9	119.8 (5)	C24—C23—H23	120.2
C11—C10—H10	120.1	N3—C24—C23	125.1 (6)
C9—C10—H10	120.1	N3—C24—H24	117.4
C10—C11—C12	119.1 (5)	C23—C24—H24	117.4
C10—C11—H11	120.4	N3—C25—C26	125.4 (5)
C12—C11—H11	120.4	N3—C25—H25	117.3
C13—C12—C11	120.9 (5)	C26—C25—H25	117.3
C13—C12—H12	119.6	C25—C26—C22	119.2 (5)
C11—C12—H12	119.6	C25—C26—H26	120.4
C12—C13—C14	120.1 (5)	C22—C26—H26	120.4
C12—C13—H13	119.9		
			
C8—N1—N2—C15	162.2 (6)	C12—C13—C14—C9	2.1 (9)
C6—C1—C2—C3	0.0	C10—C9—C14—C13	2.5 (9)
C7—C1—C2—C3	178.9 (4)	C8—C9—C14—C13	−177.5 (5)
C1—C2—C3—C4	0.0	N1—N2—C15—C16	177.4 (6)
C2—C3—C4—C5	0.0	N2—C15—C16—C21	−6.3 (11)
C3—C4—C5—C6	0.0	N2—C15—C16—C17	178.1 (7)
C4—C5—C6—C1	0.0	C21—C16—C17—C18	−0.7 (11)
C2—C1—C6—C5	0.0	C15—C16—C17—C18	175.0 (7)
C7—C1—C6—C5	−178.9 (4)	C16—C17—C18—C19	−1.2 (11)
C2—C1—C7—O1	−5.1 (6)	C17—C18—C19—C20	2.8 (10)
C6—C1—C7—O1	173.8 (4)	C17—C18—C19—C22	179.1 (6)
C2—C1—C7—C8	173.7 (3)	C18—C19—C20—C21	−2.6 (9)
C6—C1—C7—C8	−7.5 (6)	C22—C19—C20—C21	−178.9 (5)
N2—N1—C8—C9	−178.8 (5)	C19—C20—C21—C16	0.7 (10)
N2—N1—C8—C7	−9.0 (8)	C17—C16—C21—C20	0.9 (10)
O1—C7—C8—N1	−84.7 (7)	C15—C16—C21—C20	−174.6 (6)
C1—C7—C8—N1	96.5 (6)	C20—C19—C22—C23	−29.9 (9)
O1—C7—C8—C9	85.3 (6)	C18—C19—C22—C23	153.9 (6)
C1—C7—C8—C9	−93.5 (6)	C20—C19—C22—C26	148.4 (6)
N1—C8—C9—C10	0.1 (8)	C18—C19—C22—C26	−27.8 (9)
C7—C8—C9—C10	−170.0 (5)	C26—C22—C23—C24	−2.2 (9)
N1—C8—C9—C14	−179.9 (6)	C19—C22—C23—C24	176.3 (6)
C7—C8—C9—C14	10.0 (8)	C25—N3—C24—C23	−2.9 (11)
C14—C9—C10—C11	−4.3 (9)	C22—C23—C24—N3	3.2 (11)
C8—C9—C10—C11	175.7 (5)	C24—N3—C25—C26	1.9 (10)
C9—C10—C11—C12	1.6 (9)	N3—C25—C26—C22	−1.2 (10)
C10—C11—C12—C13	3.0 (10)	C23—C22—C26—C25	1.3 (9)
C11—C12—C13—C14	−4.9 (10)	C19—C22—C26—C25	−177.2 (6)

### Hydrogen-bond geometry (Å, °)

**Table:**

*D*—H···*A*	*D*—H	H···*A*	*D*···*A*	*D*—H···*A*
C26—H26···O1^i^	0.95	2.57	3.502 (7)	166

Symmetry codes: (i) −*x*+1, −*y*+1, −*z*+1.
